# Supplementary figures and images for: Integrated Omics Analysis Reveals Key Pathways in Cotton Defense against Mirid Bug (Adelphocoris suturalis Jakovlev) Feeding
Source: Insects. 2024 Apr 8;15(4):254. doi: 10.3390/insects15040254 (PMC11049813; doi:10.3390/insects15040254)

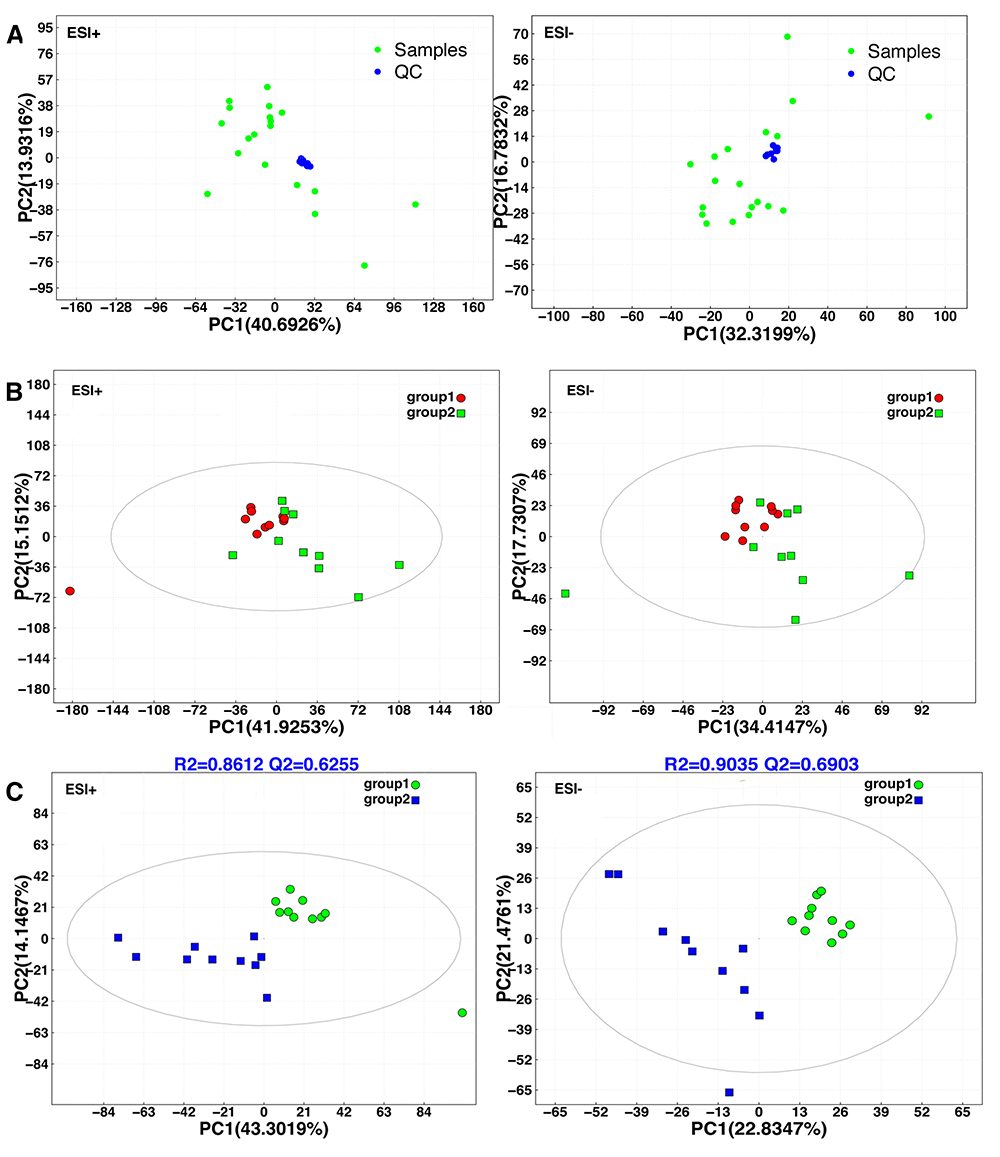

Supplement: Supplementary file 1 [file insects-15-00254-s001.zip › insects-2894859-supplementary/supplementary materials/Figure S1.tif]
